# 金属有机框架分子印迹材料的应用进展

**DOI:** 10.3724/SP.J.1123.2023.03005

**Published:** 2023-08-08

**Authors:** Wei LIU, Dongxue JIA, Wenhui LIAN, Yu ZHAO

**Affiliations:** 1.长春中医药大学药学院,吉林 长春 130017; 1. College of Pharmacy, Changchun University of Chinese Medicine, Changchun 130017, China; 2.长春中医药大学吉林省人参科学研究院,吉林 长春 130017; 2. Jilin Ginseng Academy, Changchun University of Chinese Medicine, Changchun 130017, China

**Keywords:** 金属有机框架, 分子印迹技术, 催化, 样品前处理, 药物载体, 荧光传感器, metal-organic frameworks (MOFs), molecular imprinting technology, catalysis, sample preparation, drug carriers, fluorescent sensors

## Abstract

分子印迹聚合物具有预定性、识别性和实用性等优点,受到各领域的广泛关注。但传统制备方法包埋法具有识别速率慢、位点不均匀、结合容量低、模板分子洗脱不彻底等缺点,限制了分子印迹技术的发展。为了有效解决上述问题,表面分子印迹合成策略应运而生。随后各种不同的材料作为载体应用于表面印迹聚合物的合成中,其中金属有机框架(MOFs)作为载体表现出极大的潜力。金属有机框架因具有较大的空隙率和较高的比表面积,可以为分子印迹提供大量的印迹位点,有利于提高检测的灵敏度。同时,由于金属中心和有机配体可变,还可以通过调节孔径及引入功能基团等手段,合成不同结构及性能的金属有机框架材料。金属有机框架与分子印迹技术相结合制备的材料同时兼具分子印迹技术特异性吸附和金属有机框架比表面积大、活性位点多的优势,扩大了材料的应用范围。本文从引入特殊配体、调控金属中心位点、生成MOFs复合物、MOFs衍生化、缺陷的MOFs设计等方面介绍了金属有机框架功能化的策略,并综述了MOFs基分子印迹材料在催化、样品前处理、药物载体、荧光传感器和电化学传感器领域的应用,并对MOFs基分子印迹材料存在的问题及未来发展进行了讨论与展望。

## Abstract (en)

Molecularly imprinted polymers have received wide attention from various fields owing to their pre-designable, recognition ability, and practicality. However, the disadvantages of the traditional embedding method, which include a slow recognition rate, uneven site recognition, low binding capacity, and incomplete template molecule elution, limit the development of molecular imprinting technology. Surface molecular imprinting techniques have been developed to effectively solve these problems, and different materials are used as carriers in the synthesis of molecularly imprinted polymers. Metal-organic frameworks (MOFs) show great potential as carriers. Because of their high porosity and specific surface area, MOFs can provide a large number of active sites for molecular imprinting, which can improve their detection sensitivity. The variable metal centers and organic ligands of MOF materials can also lead to multiple structures and functions. Numerous types of MOF materials have been synthesized, and the properties of these materials can be tailored by adjusting their pore size and introducing functional groups. MOFs and molecular imprinting technology can be combined to take full advantage of the specific adsorption of molecular imprinting technology and the large specific surface area and multiple active sites of MOFs, thereby expanding the application range of the resulting materials. In this paper, five aspects of the concept of MOF functionalization are discussed: introduction of special ligands, regulation of metal central sites, formation of MOF complexes, derivatization of MOFs, and sacrificial MOFs. The applications of MOF-based molecularly imprinted materials in catalysis, sample pretreatment, drug carriers, fluorescence sensors, and electrochemical sensors are also reviewed. Finally, the existing problems and future development of MOF-based molecularly imprinted materials are discussed and prospected.

分子印迹技术(molecular imprinting technology, MIT)是在分子印迹聚合物(molecularly imprinted polymers, MIPs)中构建模板分子的选择性识别位点,以达到对模板分子进行靶向识别的技术。自20世纪70年代发现以来,MIT经过了几十年的发展,现已广泛应用于样品前处理、催化、药物递送及传感领域^[1⇓⇓⇓-5]^。

MIPs的制备过程如下:通过氢键、离子键、疏水等共价或非共价相互作用进行预聚合,在交联剂和致孔剂存在下,形成聚合物;然后通过破坏印迹分子与功能单体的结合键将模板分子去除,得到对目标物具有特异性识别和结合位点的分子印迹聚合物。MIPs的常用制备方法有包埋法和表面分子印迹法^[6,7]^。包埋法简单、易操作,被广泛应用于分子印迹合成过程中。但传统的包埋法存在识别速率慢、位点不均匀、结合容量低、模板分子洗脱不彻底等不可避免的缺点。表面分子印迹法是指在某种固相的载体基质表面形成聚合物的技术,通过表面分子印迹技术合成的聚合物,其识别位点大多位于聚合物的表面,因此具有更高的吸附容量、更快的传质速率和更强的结合动力。不同的载体会赋予印迹材料不同的性能,根据文献调研可知,用于表面分子印迹合成载体的材料有活性硅胶^[8]^、磁性纳米颗粒^[9]^、壳聚糖^[10]^、量子点^[11]^和金属有机框架(metal-organic frameworks, MOFs)^[12]^等。

MOFs因具有大孔隙率和高比表面积等优良性能,已成为分子印迹技术中的常用载体。在其表面合成较薄的印迹壳层,印迹位点位于材料表面易于洗脱和吸附。由于金属中心和有机配体可变,还可以通过调节孔径及引入功能基团等手段,合成不同结构及性能的金属有机框架材料^[13,14]^。MOFs与MIT结合可以有效解决印迹材料结合容量低、识别速率慢、识别位点少、模板泄露等问题,极大地提高了MOFs材料的效率和选择性,同时也扩大了材料的应用范围。

近年来,已有多篇文章综述了分子印迹材料的现状、热点和趋势^[15⇓⇓⇓-19]^,我们课题组综述了分子印迹技术在蛋白质翻译后修饰分析中的应用^[20]^, MOFs基分子印迹材料应用方面的综述未见报道。Çorman等^[21]^重点介绍了分子印迹技术与MOFs结合如何提高电化学传感器的灵敏度及专属性,同时还评估了纳米粒子、碳基材料和具有特定功能的纳米复合材料对电化学传感器性能的影响。本文系统综述了MOFs基分子印迹材料的功能化策略及在催化、样品前处理、药物载体、荧光传感器和电化学传感器领域的应用进展,并对其未来发展做出展望,希望为MOFs基分子印迹材料的研究提供一定的参考价值。

## 1 MOF基材料

### 1.1 MOF基材料的介绍

MOFs是一类通过配位键将过渡金属离子和有机配体连接起来,经过一系列自组装行为形成的新型多孔材料。根据采用的金属和有机配体不同,可形成多种拓扑结构。最具代表性的MOFs材料分为网状金属有机框架(IRMOFs)、类沸石咪唑框架(ZIFs)、莱瓦希尔框架(MILs)、锆基金属有机框架(UiOs)及铜基金属有机框架(HKUST-1)等几类^[22]^。其中多以富马酸、2-甲基咪唑、对苯二甲酸、三甲基酸等为有机配体,以Mn、Zn、Mg、Fe、Cu和Zr等为金属中心制备得到^[23⇓⇓⇓-27]^。

### 1.2 MOF基材料的功能化

MOFs的结构具有可设计性以及易于修饰等优势,研究者采用不同的手段和方法对MOFs材料进行功能化,从而满足不同应用领域中对材料的特定要求^[28,29]^。近年来,针对MOFs基分子印迹材料功能化的策略主要分为以下几类。

#### 1.2.1 引入特殊配体

在MOFs的合成中,通过引入特定官能团的特殊配体,对其进行功能化预设计,可合成具有特定功能、稳定性好和孔隙率高的MOFs,为MOFs在不同领域的应用提供了一种实用、合理的思路。

Lei等^[30]^合成了不同有机配体的Ni基二维导电MOF(CMOFs),并研究了Ni基CMOFs的电化学性能,构建了以电导率最高、有效表面积最大的Ni_3_(HITP)_2_为载体,离子液体(IL)为单体和交联剂的电化学分子印迹传感器(CMOF-MIPIL),用于双酚A的检测,检出限为4.0 nmol/L,线性范围为0.005~5.0 μmol/L。

Yang等^[31]^采用2-氨基对苯二甲酸作为配体,合成了氨基改性的MOF(NH_2_ -MIL-125(Ti)),然后与TiO结合生成了NH_2_ -MIL-125(Ti)-TiO_2_光电化学复合物,构建了分子印迹光电化学传感器。NH_2_ -MIL-125(Ti)不仅保留了MOFs的本身特性,而且还具有出色的光致发光和光催化活性,在光电化学领域具有潜在的应用价值。

Du等^[32]^以2-氨基对苯二甲酸作为配体,合成了氨基改性的MOF(NH_2_-MIL-53(Al)),构建了荧光探针用于小麦中玉米赤霉烯酮的高选择性检测。MOF中改性基团氨基不仅通过氢键与目标物结合,而且向MOF的金属中心Al提供电子,导致MOF发出荧光。

Yahyapour等^[33]^以新型高度对称的吡啶羧酸类试剂(2,6-吡啶二羧酸铵)为配体,合成了Co-MOFs。构建了以Co-MOFs/聚乳酸(PLA)为基质的分子印迹电化学传感器用于检测环丙沙星。

Han等^[34]^以二氨基、对苯二甲酸为配体,合成了氨基和苯硼酸基团修饰的MOF(UiO-66),用以加载抗癌药物阿霉素(DOX),然后在合成的纳米颗粒表面进一步制备以唾液酸(SA)为模板的分子印迹聚合物(UiO-66-DOX@MIP)。该方法制备了一种不含抗体修饰的药物递送系统,可以有效识别肿瘤部位并控制药物递送,这对于癌症治疗至关重要。

#### 1.2.2 调控金属中心位点

为了提高MOFs对目标物的吸附、催化等作用,可通过调控金属中心位点以达到应用研究的目的。

Erk等^[35]^制备了含有Ni(Ⅱ)、Co(Ⅱ)两种金属中心的MOF(Co/Ni@MOF),双金属MOF不仅具有较高的孔隙率、更强的热稳定性,与单金属Ni@MOF和Co@MOF相比,还具有更好的电子导电性和更多的活性位点。

Amirzehni等^[36]^利用合成的双金属有机框架(CoZn ZIF)的催化性能,设计了一种选择性比色探针用以检测乐果。结果表明,CoZn ZIF的催化性能要高于单金属有机框架Co(ZIF-67)或Zn (ZIF-8)。

Fu等^[37]^制备了以稀土阳离子La为中心金属的La-MOF,将Au功能化的La-MOF封装在TiO_2_纳米颗粒中,首次构建了紫外自启动聚合传感器,实现了紫外条件下咖啡酸的特异性识别和消除。La-MOF具有独特的光致发光特性,可应用于光催化和光传感领域。

Afshar等^[38]^以Zr为中心原子、对苯二甲酸为配体合成了具有荧光特性的MOF。构建了一种新型荧光探针,用以检测辅助治疗新冠的药物褪黑激素。

#### 1.2.3 MOFs复合物

通过选择合适的制备方法,将MOFs与金属纳米颗粒、碳族材料、离子液体和多金属氧酸盐进行复合,制备了多种MOFs复合物。MOFs复合物整合了各材料间的优点,改善了各自的缺点,不仅保留了单相材料的优良性能,甚至还生成了新的特性^[39,40]^。

(1)与金属纳米颗粒复合。金属纳米颗粒具有良好的传热性和导电性,以及优异的延展性和柔韧性,作为传感元件被广泛应用于电化学传感器中。Bagheri等^[41]^通过在片状Zn基MOF的纳米孔内生成银纳米颗粒(AgNPs),合成了复合材料AgNPs@ZnMOF,以该复合材料作为分子印迹聚合物的有效载体,成功用于棒曲霉素的选择性测定。Mahnashi等^[42]^采用氮掺杂碳纳米管、银纳米颗粒改性的MOF(Cu-BDC)修饰玻碳电极,构建了电化学传感器,成功用于测定药物注射剂、人血浆和尿液样品中的抗癌药奥沙利铂。Ag改性的MOF可有效改善MOF因导电和机械稳定性差而带来的缺陷。Liu等^[43]^采取原位生长法在纳米Cu_2_O芯材上合成了HKUST-晶体,并利用置换反应将金纳米颗粒封装在中空的HKUST-1壁内,制备了Au@HKUST-1纳米胶囊,该胶囊表现出较高的电催化活性,并有效改善了电化学信号。

(2)与碳族材料复合。MOFs机械稳定性和导电性均较差的特性极大地限制了它在电化学领域的应用,但与介孔碳(MC)、多壁碳纳米管、介孔二氧化硅等碳族材料相结合,可有效缓解上述问题。Rawool等^[44]^提出了一种CuMOF/MC/MIP分子印迹电化学传感器,用于对利福平和异烟肼同时进行高灵敏度、高选择性检测。介孔碳的引入有效弥补了Cu-MOF导电性、机械稳定性和热稳定性较差的缺点。Lu等^[45]^制备了羧酸多壁碳纳米管(c-MWCNTs)和ZIF复合材料,构建了c-MWCNTs-ZIF分子印迹电化学传感器,用以检测呋喃西林。c-MWCNTs的引入有效弥补了MIP低电导率的缺陷,同时也大大提高了传感器的灵敏度。Sun等^[46]^通过在介孔二氧化硅纳米球(MSN)载体的外表面上生长ZIF-8纳米晶,合成了花状形貌的核壳型复合物,该复合物在吸附性能、催化活性和反应速率方面表现出优异的协同作用。

(3)与离子液体复合。离子液体具有不易挥发、液态温度范围宽、稳定性好、溶解能力强、可通过结构的改变调节其理化性质以及可以循环利用等性质,与MOFs复合后生成的材料表现出极强的富集能力。Luo等^[47]^制备了钴金属有机框架(Co MOF)-离子液体纳米复合材料,以其为基质构建分子印迹电化学传感器,用以检测乳腺癌的生物标志物癌胚抗原(CEA)。离子液体的添加可以显著提高电信号的响应灵敏度,促进电化学中氧化还原反应的电子转移。

(4)与多金属氧酸盐复合。多金属氧酸盐(POMs)因具有优异的氧化还原性能而被广泛用作催化剂,但因POMs比表面积较小,在实际应用中,催化活性位点得不到充分的利用,将POMs固载到MOFs上,POMs呈高度分散状态,有效改善其传质性能。Huang等^[48]^采用一步水热合成法制备了磷钼酸MOF复合材料(PMo_12_@UiO-66),并以此复合材料为载体,在其表面制备了具有核壳结构的吸附材料PMo_12_@UiO-66@H_2_S-MIPs,实现了对H_2_S气体的选择性吸附,吸附容量为24.05 mg/g。

#### 1.2.4 MOFs衍生化

由于MOFs不具备良好的导电性和稳定性,研究者通常采用高温煅烧的方法获得MOFs碳化衍生材料,该材料既保留了MOFs结构的多孔性和多样性,又有效地增强了其稳定性与导电性能。

Cheng等^[49]^将银纳米粒子(AgNPs)负载在MOF(Cr-MIL-101)表面上,并引入生物质碳(BC)后进行高温煅烧,合成了纳米复合材料(BC/Cr_2_O_3_/Ag)。该复合材料不仅保持了框架结构的大比表面积,而且还提高了电导率以增强电流响应。Bu等^[50]^通过煅烧UiO-66合成了纳米多孔碳材料ZrO_2_@C。ZrO_2_纳米粒子均匀地分布在多孔碳中,并与碳紧密结合。与未经衍生化处理的UiO-66相比,ZrO_2_@C的导电性得到了显著地增强。Duan等^[51]^首次以钴(Ⅱ)离子交换的锌基生物金属有机框架(Zn-bio-MOF-1)为前驱体,采用溶剂热法合成了CoS纳米颗粒附着的ZnS棒(CoS/ZnS复合材料)。该复合材料保留了Zn-bio-MOF-1的高比表面积和丰富孔隙率,为电化学反应提供了大量的活性位点。

#### 1.2.5 缺陷的MOF设计

为增大孔径、引入额外的吸附位点、改善表面积,研究者在合成MOFs时通常在前驱混合物中加入调制剂,生成有缺陷的MOFs。

Iskierko等^[52]^利用缺陷MOF-5设计了一种用于测定中性粒细胞明胶酶相关脂质运载蛋白的高灵敏度、高选择性的电化学传感器。

## 2 金属有机框架基印迹材料的应用

### 2.1 催化

MOFs具有比表面积大、多孔结构可调节、活性位点丰富等特点,可有效增强催化剂的催化性能。近几年,研究人员热衷于MOFs基材料的催化作用探索^[53⇓-55]^。

Chen等^[56]^以MOF作为基质固定葡萄糖氧化酶(GOx)和牛血红蛋白(BHb),合成了级联催化体系(MOF@GOx@BHb)。分别以*β*-D-葡萄糖、甘露糖、木糖和半乳糖为模板,在受限的酶促系统中连续生成羟基自由基,用以引发MOF@GOx@BHb表面的印迹聚合反应的发生,制备的印迹聚合物(MOF@GOx@BHb@MIPs)对目标物实现了特异性催化,在食品安全中发挥极其重要的作用。

Ding等^[57]^将Fe-MOF-74与MIP结合,采用原位生长法制备了靶向催化剂(Fe-MOF-74/MIP),实现了邻苯二甲酸二甲酯(DMP)的高效降解。为了进一步提高催化剂的水稳性和催化活性,Ding等^[58]^还制备了核壳型催化剂(Fe-MOF-74@SiO_2_@MIP),循环实验表明,该催化剂在水溶液中具有强稳定性和可重复利用性。此外,在模拟实际水处理实验中,在保持初始pH为7不变的情况下,DMP仍然会被有效降解,表明该催化剂具有潜在的应用价值。

Bagheri等^[41]^将合成的AgNPs@ZnMOF复合材料作为对苯二甲酸(TA)-H_2_O_2_反应的高效过氧化物酶样催化剂,利用棒曲霉素可显著降低AgNPs@ZnMOF的催化活性,设计了一种对棒曲霉素具有超灵敏检测的荧光探针。此外,Bagheri等^[59]^合成了双金属ZnCo MOFs,并对其催化性质进行研究,实验结果表明,与ZIF-8和ZIF-67相比,ZnCo MOFs具有优异的催化活性。鉴于阿托品会降低ZnCo MOFs的催化活性,他们建立了一种MIP预提取和荧光检测系统相结合的方法,成功用于血清中阿托品的检测。

### 2.2 样品前处理

MOFs和MIPs都已被广泛用于样品前处理中^[60,61]^,但两者又具有各自的缺点。MOFs与目标物的作用机理主要依靠氢键、*π-π*、疏水和静电等相互作用,因此其选择性较差。而传统的分子印迹合成方法得到的MIPs具有模板渗漏、形状不规则以及耗时费力等缺点。近几年,研究者致力于将MOFs和MIPs联用,探索其在样品前处理领域的应用。

Rahimpoor等^[62]^合成了一种高效多孔吸附剂MOF@MIP,并成功利用针头捕集装置对二嗪农药进行取样、提取和测定。该方法简单、快速、环保、选择性高,对空气中二嗪酮的采集和测定具有潜在的应用价值。

陈颖等^[63]^以MOF199为基体,制备了新型表面分子印迹聚合物材料,用以对二苯并噻吩进行靶向吸附,吸附量为37.79 mg/g, 印迹因子(IF)=2.29,吸附行为遵循伪一阶动力学模型,说明吸附主要为物理过程。

Alilou等^[64]^开发了一种以磁性氧化石墨烯和锆金属有机框架为基底的纳米分子印迹吸附剂(MGO/MOF-808@MIP),成功用于黄曲霉毒素的提取和预浓缩。

Wei等^[65]^报道了特异性识别*S*-氨氯地平(*S*-AML)的核壳型有机-无机杂化分子印迹聚合物。以金属有机框架MOF-177为核,以*S*-AML为模板,甲基丙烯酸为功能单体,四乙氧基硅烷为交联剂,乙酸为催化剂,在MOF-177表面生成了印迹壳层。该印迹材料获得了更快的结合动力学及传质速率,能高选择性识别*S*-AML(IF=5.79),吸附容量达到1.31 mmol/g。

Li等^[66]^结合表面印迹技术,以MOF-74(Ni)为基质,溶菌酶为模板制备了溶菌酶印迹聚合物(MOF@PDA-MIP)。MOF-74具有较大的比表面积(150.0 m^2^/g),为聚合物层提供了更多的识别位点;MOF-74(Ni)中的Ni^2+^与蛋白质之间的螯合作用有利于溶菌酶固定在MOF@PDA-MIP的表面。MOF@PDA-MIP对溶菌酶表现出高效及特异性识别能力,10 min内即可达到吸附平衡,最大吸附容量为313.5 mg/g, IF=7.8。

Wang课题组在分子印迹聚合物的合成方面积累了丰富的经验^[67⇓⇓-70]^。课题组的Huang等^[48]^报道了一种对H_2_S选择性吸附的分子印迹材料。为了摆脱CO_2_的干扰,以磷钼酸PMo_12_和UiO-66生成的复合物PMo_12_@UiO-66为载体,H_2_S为模板分子,丙烯酰胺为功能单体,乙二醇二甲基丙烯酸酯(EGDMA)为交联剂,过氧化苯甲酰和*N*,*N*-二甲基苯胺为引发剂,制备了核壳结构的H_2_S印迹材料(PMo_12_@UiO-66@H_2_S-MIPs)。该印迹材料与非印迹材料(PMo_12_@UiO-66@H_2_S)相比,脱硫效率更好,吸附量更高(24.04 mg/g)。同时考察了该印迹材料对H_2_S和CO_2_的选择性吸附,在CO_2_的干扰下,印迹材料对H_2_S具有高选择性吸附,吸附量也没有降低。

Wan等^[71]^建立了一种咪唑离子液体功能化的金属有机框架(ZIF-67)分子印迹材料固相萃取(ZIF-67@[Bmim][Br]@MIP)双酚A的方法。并将该方法成功用于矿泉水瓶、牛奶盒和奶茶杯中双酚A的检测。

Asfaram等^[72]^利用金属有机框架(HKUST-1)作为载体合成了表面分子印迹聚合物,作为磁性固相萃取材料与紫外可见光谱仪联用,用于检测没食子酸。该方法线性范围宽(8~6000 ng/mL)、检出限低(1.377 ng/mL)、回收率良好(98.13%)。

Firoozichahak等^[73]^以合成的核壳型磁性金属有机框架(Fe_3_O_4_@SiO_2_@MIL-101 (Fe))作为基底,在其表面印迹聚合,制备了分子印迹聚合物(Fe_3_O_4_@SiO_2_@MIL-101 (Fe)@MIP),该印迹聚合物与气相色谱联用,实现了尿液样品中二嗪农药的高选择性、快速、灵敏的检测分析。

Zhang等^[74]^采用了一种简单有效的逐层合成策略,制备了核壳型磁性金属有机框架分子印迹复合物(Fe_3_O_4_@ZIF-8@MIP),该复合物作为磁性固相萃取材料与高效液相色谱联用,对食品样品中的双酚A进行检测。实验结果表明,Fe_3_O_4_@ZIF-8@MIP对食品中的双酚A表现出良好的识别能力。

Bagheri等^[41]^首次报道了一种具有过氧化物酶样活性的MOF纳米复合材料(AgNPs@ZnMOF)作为分子印迹聚合物的有效载体,成功用于棒曲霉素的选择性测定。

### 2.3 药物载体

MOFs可以通过氢键或离子静电与药物相互作用,从而达到稳定的药物释放效果^[75⇓-77]^。但MOFs作为药物载体,存在载药量低、缺乏特异性识别等缺点。将MOFs与MIPs结合作为药物载体用于药物递送,可有效解决上述问题。

Zhang等^[78]^制备了一种液晶分子印迹聚合物,用于卡培他滨(CAPE)的缓释载体。MOF(HKUST-1)因其孔体积大、扩散性好、热稳定性高而被作为载体,在其表面印迹了液晶MIP (HKUST-1@LC-MIP)。体内外药物释放实验表明,HKUST-1@LC-MIP对CAPE的控释效果很好,使CAPE在大鼠体内具有较高的相对生物利用度。Han等^[34]^合成了基于氨基和苯硼酸基团修饰UiO-66的分子印迹聚合物(UiO-66-DOX@MIP)。该聚合物作为载体可以特异性识别唾液酸,并向前列腺癌细胞DU 145实现靶向递送DOX。印迹层可以有效防止正常体液中的药物泄漏,并在肿瘤微环境中达到药物释放的目的。UiO-66-DOX@MIP具有优异的生物相容性,在癌症治疗和靶向给药中具有良好的应用前景。Shi等^[79]^报道了一种基于MOF(MIL-101)的分子印迹纳米复合材料,用于DOX的高选择性识别和递送。吸附实验表明,MIPs对DOX负载具有较高的灵敏度、选择性和优越的吸附效率。在pH 5.5和7.4下,合成的药物载体对DOX的72 h累积释放率分别为46.19%和28.03%。细胞毒性试验结果表明,MIPs对肝癌细胞(HepG2)和正常肝细胞(HL-7702)的抑制率分别为30.1%和23.93%。鉴于该分子印迹聚合物具有优异的生物相容性、载药量和缓释性能,未来有望成为缓释药物体系中很有前景的载体。

### 2.4 荧光传感器

MIPs因特异性吸附被广泛应用于分析检测领域,但因MIPs不具备信号传输性能,若构建荧光传感器,需要引入荧光材料。将荧光信号的高灵敏性能与MIT的高选择性能相结合,来实现分子印迹荧光传感器对目标物的选择性识别与高灵敏检测。我们课题组在功能化金属有机框架及发光金属有机框架荧光传感器的构建中积累了丰富的经验^[80⇓-82]^。

MOFs具有的杂化结构和多样性的光学性质,进一步推动了荧光MOFs在分析检测中的应用^[83⇓⇓-86]^。Zhang等^[87]^以对苯二甲酸(PTA)为配体,合成了MIL-53(Fe),通过简单的自聚合将聚多巴胺(PDA)涂覆在MIL-53(Fe)表面,制备了具有增强过氧化物酶样活性和特异性识别甲硝唑(MNZ)的荧光传感器(MIL-53(Fe)@MIP)。MIL-53(Fe)的发光来源于对苯二甲酸(PTA)捕获羟基自由基生成羟基对苯二甲酸(PTA-OH), PTA-OH可发出蓝色荧光。应用该传感器对MNZ进行了定量检测,线性范围为1~200 μmol/L,检出限为53.4 nmol/L。该传感器还应用于实际样品牛奶和人血清中MNZ的检测,回收率为93.2%~102%, RSD低于3%。Eskandari等^[88]^以CP为模板,在发光的金属有机框架(MOF-76)表面生成了介孔分子印迹聚合物(mMIP)层,构建了用于检测毒死蜱的高度特异性荧光探针。棒状MOF-76的高孔隙率以及MIP层的介孔结构极大地改善了分子的扩散过程,提高了探针的灵敏度。Fu等^[37]^报道了一种基于Au和TiO_2_共功能化的镧系金属有机框架(La-MOF)的紫外自引发聚合传感平台,用于咖啡酸(CA)的特异性识别和消除。该聚合反应的发生是由TiO_2_在紫外光照射下产生的羟基自由基(·OH)引发的,不需要额外引发剂的加入,这比传统的MIT更环保,更高效。同时,La-MOF可以发出荧光以辅助TiO_2_在紫外光下的光催化,而金纳米粒子(AuNPs)可以分解TiO_2_光生成的过氧化氢,导致·OH的连续生成,并形成了高效的级联反应体系。该传感器成功用于CA的特异性检测,检测范围为1~150 μmol/L,检出限为0.15 μmol/L。

另外,虽然有些MOFs本身不能发出荧光,但可通过后续内嵌发光染料或功能化荧光纳米粒子,来构建荧光传感器^[89]^。Bagheri等^[41]^报道了一种基于AgNPs@ZnMOF的分子印迹荧光探针,用于在复杂培养基中超灵敏、高选择性测定棒曲霉素的含量。Xu等^[90]^采用一锅法制备了碳点嵌入金属有机框架的分子印迹光感器(CDs@MOF@MIP),用于选择性检测银杏叶胶囊中槲皮素的含量,浓度范围为0~50.0 μmol/L, 检出限为2.9 nmol/L。Pirot等^[91]^提出了一种对抗坏血酸(AA)具有高选择性的比率荧光探针。在纳米尺寸的ZIF-8上生成分子印迹层,以蓝色铜纳米簇(B-CuNCs)和黄碳点(Y-CDs)为荧光信号转换器(MIP@ZIF-8),实现了对AA的荧光检测。该探针对AA的检出限为1.56 μmol/L,线性范围为4.0~22.0 μmol/L。

近四年来,Cai课题组设计了系列用于病毒检测的分子印迹传感器^[92]^。该课题组首先提供了一种多功能分子印迹传感器,用于单次或同时测定甲型肝炎病毒(HAV)和乙型肝炎病毒(HBV)^[92]^。由于绿色和红色量子点的颜色随目标物质浓度的变化而变化,实现了视觉检测。亲水性单体和金属螯合作用的结合降低了非特异性结合,增强了吸附的特异性。该传感器对两种病毒检测的选择性和灵敏度均达到令人满意的结果,20 min内即可完成测定,HAV和HBV的印迹因子分别为3.70和3.35,检出限分别为3.4和5.3 pmol/L。随后该课题组提出了一种用于检测HAV的pH响应型分子印迹传感器^[93]^。以MOF(MIL-101)为基底,在其表面生成印迹聚合物。质子化的基团Me_2_N与DMA侧链之间相互排斥,该印迹聚合物会随着pH的变化而膨胀,有利于模板的洗脱,20 min内即可完成HAV的检测,线性范围为0.02~2.0 nmol/L,检出限为0.1 pmol/L。紧接着该课题组报道了一种基于金属有机框架的荧光分子印迹传感器,以丙烯酸锌为功能单体,在硅改性的金属有机框架表面上通过自由基聚合法生成了分子印迹聚合物,将聚乙二醇(PEG)用作封闭剂,以增强聚合物特异性识别模板病毒的能力。该传感器对日本脑炎病毒(JEV)表现出高选择性、高灵敏度的识别能力,在20 min内即可完成对JEV的检测,检测范围为50~1400 pmol/L,检出限为13 pmol/L, IF=4.3^[94]^。2021年,该课题组又报道了一种基于磁性金属有机框架的新型荧光分子印迹传感器,用于弱荧光HAV的检测^[95]^。发光MOF(MIL-101-NH_2_)既可以用作输出信号,也可以用作载体,代替了传统的量子点和有机染料,用来产生荧光输出信号。该传感器对模板识别表现出优异的选择性以及较高的检测灵敏度,在15 min内即可完成病毒的检测。所建立的方法还成功应用于人血清样品中HAV的定性、定量分析。总之,该方法具有简单、快速、高选择性的特点,在弱荧光和没有影响的病毒检测中具有潜在的应用价值。

### 2.5 电化学传感器

电化学传感器因成本低、灵敏度高、操作简单等优点,已成为分析检测领域不可或缺的手段。分子印迹材料因具有特异性识别性能,非常适合作为敏感元件用于电化学传感器的构建。同时再将MIPs与性能优异的MOFs相结合,可构建具有高灵敏度的电化学传感器,实现了对目标物的快速分析。近些年,基于MOFs分子印迹材料构建的电化学传感器,已广泛应用于药物分析、环境检测、食品安全、临床药物监测领域(见表1)。

**表1:** 基于MOF分子印迹材料构建的电化学传感器的应用

Method of constructing electrode	Analyte	Sensor	LOD/ (mol/L)	Linear range/ (mol/L)	Samples	Ref.
In-situ polymerization	bisphenol A	CMOF-MIPIL/GCE	4.0×10^-6^	5.0×10^-9^-5.0×10^-6^	lake river	[30]
Electropolymerization	oxaliplatin	MIP-Ag@Cu-BDC/N- CNTs/GCE	0.016^*^	0.056-200^*^	pharmaceutical injections, human plasma, human urine	[42]
Molecular self-assembly	isoproturon	Au@HKUST-1@MIP	4.5×10^-10^	1.0×10^-9^-4.5×10^-5^	water	[43]
Electropolymerization	atropine	MSN@ZIF-8@MIP	9.8×10^-10^	5.0×10^-9^-9.5×10^-6^	human serum, human urine, beef, cereals	[46]
Electropolymerization	carcinoembry- onic antigen	MIP/CS/Co MOF-IL/SPCE	2.4×10^-5^	1.0×10^-4^-10^*^	human serum	[47]
Surface coating	nitrofurazone	BC/Cr_2_O_3_/Ag/MIP/GCE	3.0×10^-9^	5×10^-9^-1×10^-5^	human urine, human blood	[49]
Electropolymerization	chloroneb	MIP/CoS/ZnS	8.7×10^-10^	3×10^-9^-2×10^-7^	licorice, cucumber, river water, soil	[51]
Molecular self-assembly	furosemide	C_3_N_4_@ZIF-8@MIP@GCE	8.0×10^-9^	8.0×10^-8^-1.0×10^-4^	human urine	[96]
Surface coating	ketamine	KT-MIM/G@MOFs/SPE	4.0×10^-11^	1.0×10^-10^-4.0×10^-5^	human urine, saliva	[97]
Electropolymerization	thimerosal	Au@4-ATP@MIP	3.5×10^-14^	8.0×10^-13^-8.0×10^-11^	chloramphenicol eye drop	[98]
Electropolymerization	hygromycin B	MIP@Cu-MOF@Ti_3_C_2_T*_x_*	1.92×10^-9^	5×10^-9^-5×10^-6^	pork, chicken, fish	[99]
Surface coating	fenamiphos	MIP-Au@MOF-235@g-C_3_N_4_	7.13×10^-3^	1.0×10^-8^-1.64×10^-5^	human plasma, tomato, orange juice, lettuce	[100]
Electropolymerization	quercetin	MIP/MIL-101(Cr)/MoS_2_/ GCE	6.0×10^-8^	1.0×10^-7^-1.05×10^-5^, 1.05×10^-5^-7.0×10^-4^	honey	[101]
Electropolymerization	pregabalin	GCE/Cu-BTC/ePDA-MIP	2.9×10^-15^	5.0×10^-14^-8.0×10^-10^	human serum, human urine	[102]
Electropolymerization	clenbuterol hydrochloride and ractopamine	N@Fe-MOF@C-DTMIP	3.03×10^-13^	1.0×10^-12^-8×10^-9^	human urine, pork	[103]
Surface coating	hemoglobin	AuE/Ag-MOF@MC-MIPs	9.0×10^-14^	2.0×10^-13^-1.0×10^-6^	human blood	[104]
Molecular self-assembly	carbendazim	HKUST-1@MIP	2.0×10^-9^	1.0×10^-8^-5.0×10^-5^	apple juice, cucumber juice, tomato juice, tangerine juice	[105]
Electropolymerization	patulin mycotoxin	MIP/Au@PANI/SeS_2_@Co	6.6×10^-13^	1.0×10^-12^-1.0×10^-9^	apple juice	[106]
Electropolymerization	lidocaine	MIP/PC/GCE	6.0×10^-11^	2.0×10^-10^-8.0×10^-6^	rat serum	[107]

^*^Content unit, ng/mL.

#### 2.5.1 药物分析

研究者通常采用色谱法、质谱法等手段对药物进行分析检测,但以上方法存在仪器成本高、预处理较为复杂、操作繁琐及有机溶剂用量较多等缺点,分子印迹电化学传感器因灵敏度高、选择性强、稳定性好等特点,为药物分析提供了一种新的思路。Huang课题组^[108]^构建了以AuNPs和钴铁基-金属有机框架(CoFe-MOFs)支撑聚吡咯(Ppy)基的三明治夹心结构传感器(MIP@CoFe-MOFs@AuNPs),用于检测诺氟沙星(NOR)。经CoFe-MOFs/AuNPs修饰的MIP传感器的有效表面积是未经修饰MIP传感器的2.2倍,并成功应用于实际样品中NOR的检测。

#### 2.5.2 环境检测

随着环境中有毒有害污染物的与日俱增,人体健康受到的威胁越来越大,建立环境污染物的高效检测方法显得尤为重要。而分子印迹电化学传感器因其突出优势在众多检测方法中脱颖而出。

Gao等^[109]^利用热聚合法制备了一种用于特异性识别邻苯二甲酸二辛酯(DOP)的分子印迹光电化学传感器。MOF通过简单的浸渍法涂覆在Cu_2_O上形成异质结构,用Cu_2_O半导体和MOF的异质结构作为DOP印迹光电化学传感器的平台。该方法在实际样品分析中具有良好的灵敏度、稳定性、选择性和重现性,MOF的异质结构在光电化学分析领域具有广阔的应用前景。

Li课题组制备了一系列分子印迹复合材料^[110⇓⇓⇓-114]^。该课题组构建了一种新型IL复合的金属有机框架分子印迹电化学传感器(CMOFMIPIL),用于双酚A的高灵敏度检测^[30]^,检测范围为0.005~5.0 μmol/L,检出限为4.0 nmol/L。该方法成功用于湖水、河水中双酚A的检测。

喻派等^[115]^开发了一种能富集和快速检测印染水体中痕量偶氮染料活性艳红(K-2BP)的分子印迹电化学传感器(K-2BP-MIP@AlxFey-MOF),该传感器对K-2BP表现出特异性的识别能力。

#### 2.5.3 临床药物监测

由于临床药物基质复杂、药物浓度低,MOFs基分子印迹传感器同时具备高选择性、低检出限的优势,为临床药物监测提供了新的思路。

Cheng等^[49]^提出了一种BC/Cr_2_O_3_/Ag纳米复合材料修饰的分子印迹电化学传感器,用于呋喃西林的快速检测。首先将银纳米粒子负载在MOFs(Cr-MIL-101)上,然后引入生物炭高温煅烧合成了BC/Cr_2_O_3_/Ag纳米复合材料。来自MOF的Cr_2_O_3_/Ag不仅扩大了电极的有效表面积,而且增强了电流响应。以甲基丙烯酸和丙烯酰胺为双功能单体,呋喃西林为模板分子,在BC/Cr_2_O_3_/Ag上印迹聚合,并通过差分脉冲伏安法对呋喃西林进行检测。该传感器已成功用于人血、尿液以及药物中呋喃西林的快速检测。

Rawool等^[44]^报道了一种双模板印迹策略的电化学传感器,用于同时检测利福平(RIF)和异烟肼(INZ)。首先采用Cu-MOF与介孔碳对玻碳电极进行修饰,然后通过氢键作用将双模板分子RIF和INZ印迹薄膜沉积在电极表面。该传感器可实现在较宽的线性范围内(0.08~85 mol/L)测定RIF和INZ,其检出限分别为0.28 nmol/L和0.37 nmol/L。此外,该传感器还成功用于血清和尿液样品中RIF和INZ的同时测定。

Wang等^[96]^开发了一种C_3_N_4_/ZIF-8@MIP分子印迹电化学传感器,用于检测呋塞米。将MOF(ZIF-8)作为基底,在其表面生成印迹薄膜,之后与C_3_N_4_结合修饰玻碳电极。C_3_N_4_纳米片与ZIF-8不仅增强了传感系统的导电性,而且增加了分子印迹腔的数量。该方法成功用于呋塞米利尿剂的实时监测和兴奋剂的检测。

Fu等^[97]^采用紫外引发技术制备了一种快速检测氯胺酮的分子印迹电化学传感器。以石墨烯和金属有机框架作为基底,支撑起分子印迹聚合物。在石墨烯和MOF的共同作用下,该传感器具有较高的灵敏度,同时还具有较低的检出限(4.0×10^-11^ mol/L)和优异的选择性,并成功用于人体血液和尿液中氯胺酮的测定。

#### 2.5.4 食品安全检测

食品安全问题一直备受关注,研究者一直致力于食品安全检测方法的开发。因食品样品基质复杂,准确检测目标物具有一定的挑战性。

Hatamluyi等^[116]^报道了一种用于超灵敏检测棒曲霉素的电化学传感器,首先在电极表面修饰氮掺杂的石墨烯量子点和金纳米粒子功能化的铜-金属有机框架;然后利用原位电聚合法在其表面生长出一层分子印迹聚合物。铜-金属有机框架的独特结构为分子印迹层提供了更大的表面积,有效地增加了聚合物上的结合位点,所制备的分子印迹传感器具有宽的线性范围(0.001~70.0 ng/mL)及较低的检出限(0.0007 ng/mL)。

Gan等^[117]^报道了一种三维网络分子印迹电化学传感器,以实现对食品中茶碱的高效和特异性检测。介孔二氧化硅纳米球(MSN)用以传输电信号,以茶碱作为模板分子,苯基三乙氧基硅烷和吡咯作为功能单体,在电极表面合成了分子印迹膜。该印迹材料具有高电导率和电子转移能力,对茶碱具有优异的灵敏度和结合力,线性动态浓度范围超过6个数量级,检出限低(0.66 nmol/L),满足茶碱痕量分析的要求。

## 3 总结和展望

由于MOFs具有可变的金属中心及有机配体,并可与多种材料复合,研究者不断致力于功能化MOFs的制备及应用。本文评述了基于MOFs分子印迹材料在催化、样品前处理、药物载体及传感器领域的研究现状,并对以后的研究提出以下展望:(1)基于MOFs分子印迹材料在电化学传感器中的应用报道逐渐增多,但在荧光传感器中的研究关注度不够,应多尝试发光MOFs的荧光传感器研究,或者通过引入荧光材料,进一步提升MOFs在荧光传感器中的应用潜力。此外,在化学传感器中的应用还需进一步探索和开发用于直接定性定量分析的方法。(2)在催化领域使用的MOFs基分子印迹材料回收时一般通过高速离心分离或过滤的方式,但高速离心会造成材料损失和活性降低,因此该材料在催化领域的应用有待进一步探究。(3)由于MOFs的孔径较小,不利于大分子化合物的扩散和传质,因此MOFs在大分子化合物吸附与去除方面的应用有待突破。(4)研究者已在MOFs分子印迹材料的实际应用中做出了一些尝试,若能构建便携式传感器或商用传感器,将会极大地增加MOFs基分子印迹传感器的实用性。
